# Supplementary material for: Ethanol induces subcellular trafficking of the RNA-binding protein, hnRNP A1, in neuronal cells in vitro, but not in the peripubertal rat brain
Source: Biol Open. 2025 Jul 15;14(7):bio062010. doi: 10.1242/bio.062010 (PMC12309889; doi:10.1242/bio.062010)
Supplement: Supplementary information [file biolopen-14-062010-s1.pdf]

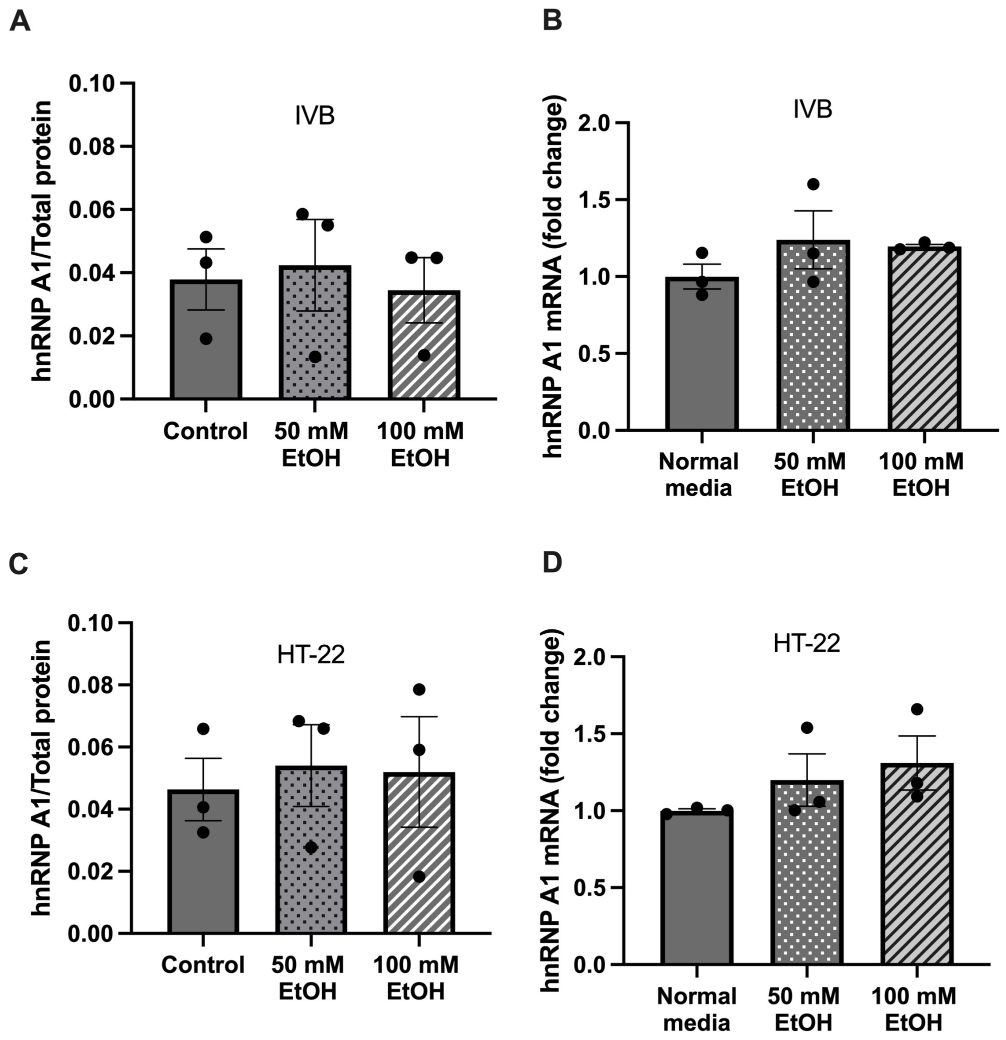

Fig. S1. EtOH did not alter hnRNP A1 mRNA and protein expression IVB cells (A, B) and HT-22 cells (C, D) were treated with normal media or media containing 50 mM EtOH or 100 mM EtOH for 2 hrs. Cells were lysed and processed for total hnRNP A1 protein expression using Western blot and mRNA using RT-qPCR. Data are depicted as mean ± SEM and one-way ANOVA for statistical analysis. N = 6 technical replicates; each assay was repeated in 3 independent experiments. No significant differences were observed.

Biology Open • Supplementary information

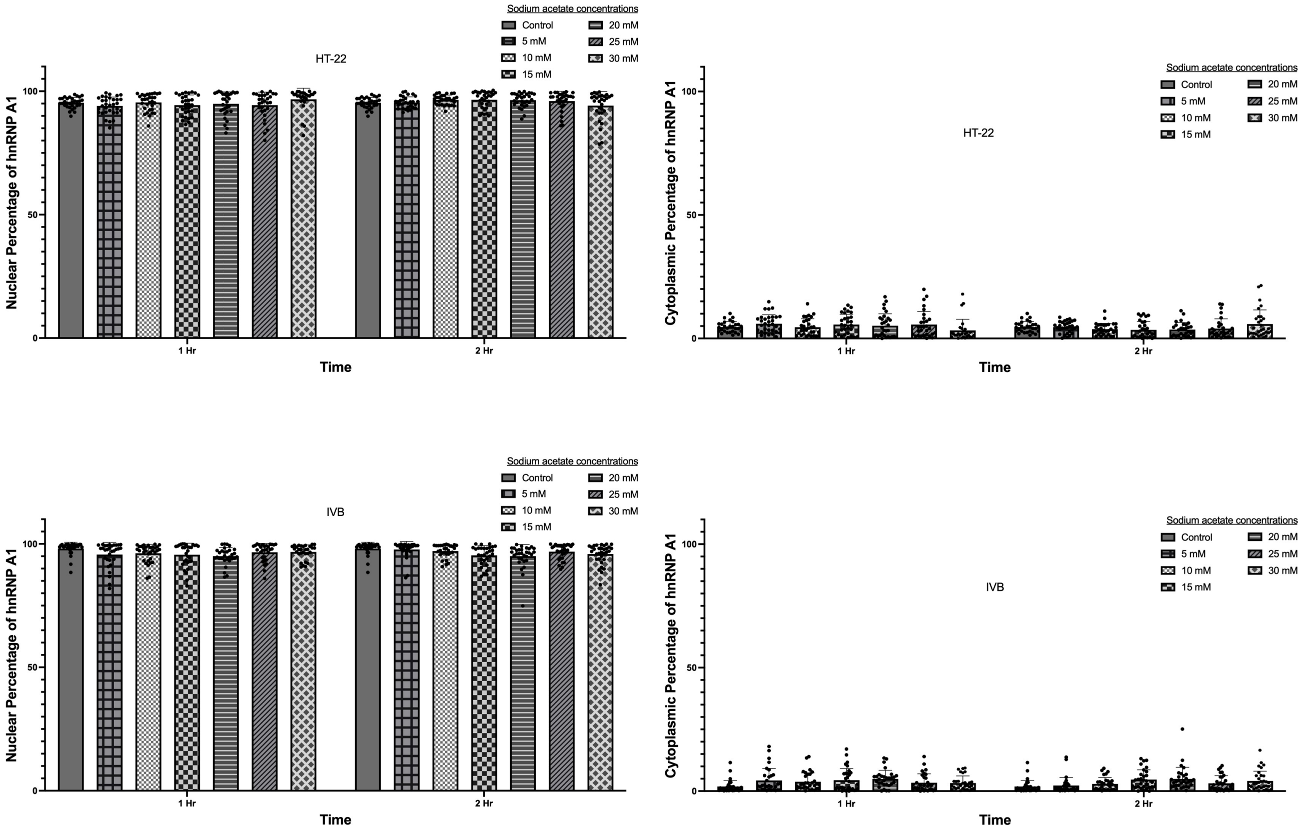

Fig. S2. Dose-dependent acetate treatment on HT-22 (top panels) and IVB (bottom panels) cells did not induce hnRNP A1 localization. Quantification of nuclear (left panels) and cytoplasmic (right panels) hnRNP A1 in HT-22 and IVB cells. Cells were treated for 1 or 2 hours with normal media or media containing varying concentrations of sodium acetate: 5 mM, 10 mM, 15 mM, 20 mM, 25 mM, or 30 mM. Data are depicted as mean ± SEM. N = 3 (6), biological (technical within each biological). No statistically significant differences were observed.

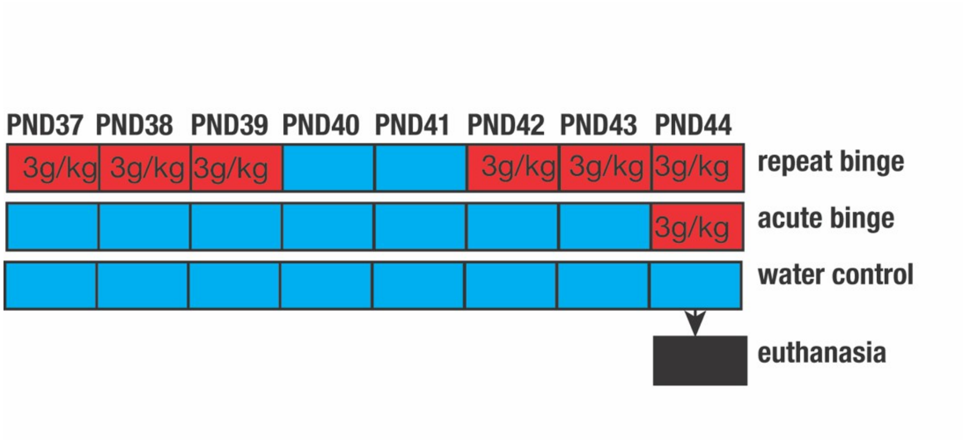

Fig. S3. Repeated binge-pattern alcohol paradigm. Peri-pubertal rats (PND 37 - 44) were given water (blue) or EtOH (red) once a day for an 8-day period at 3.2 g/kg by oral gavage. The last dosage of EtOH was given 1 hour before euthanasia.

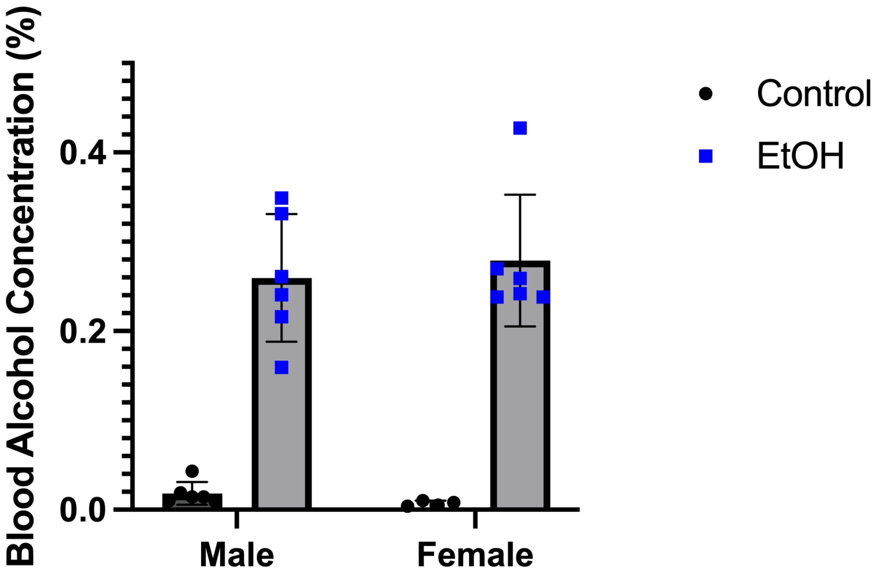

Fig. S4. Blood alcohol concentration (BAC) of peri-pubertal rats exposed to repeated binge EtOH. Following our repeated binge-EtOH exposure, peri-pubertal male and female rats reached a BAC of 0.26% and 0.28%, respectively. The control group exhibited a BAC of 0%. Each data point represents one animal per treatment, as shown in black dots (control) and blue squares (EtOH).

Biology Open • Supplementary information
